# Text mining online disinformation about antihypertensive agents ACEI/ARB and COVID-19 on Sina Weibo

**DOI:** 10.7189/jogh.13.06028

**Published:** 2023-08-18

**Authors:** Chunli Wang, Bing Fang, Aksara Regmi, Yoshifumi Yamaguchi, Ling Yang, Yuyang Cai

**Affiliations:** 1 Department of Geriatrics, Xinhua Hospital affiliated to Shanghai Jiao Tong University School of Medicine, Shanghai, China; 2 Department of Information Management, School of Management, Shanghai University, Shanghai, China; 3 School of Public Health, Shanghai Jiao Tong University School of Medicine, Shanghai, China; 4 Department of Geriatrics, Shanghai Fourth People’s Hospital affiliated to Tongji University, Shanghai, China; 5 China Institute for Urban Governance, Shanghai Jiao Tong University, Shanghai, China

## Abstract

**Background:**

The global COVID-19 pandemic outbreak has caused a significant social and economic burden, with over 4.7 million confirmed cases and thousands of casualties. Moreover, pandemic-related misinformation and disinformation on social media platforms have led to intense psychosocial issues. We investigated online disinformation about angiotensin-converting enzyme inhibitors (ACEI)/angiotensin receptor blocker (ARB) drugs and their relationship to COVID-19 on Sina Weibo.

**Methods:**

We searched for posts related to the pandemic from its beginning in December 2019 to 19 January 2021. We used text mining to identify content related to “antihypertensive agents ACEI/ARB can increase COVID-19”.

**Results:**

We found 82 posts spreading disinformation and 44 posts dispelling disinformation. The former had 535 clicks and concerns and 31 comments, and was forwarded 98 times. Of the 82 posts spreading disinformation, 15.9% (n = 13) contained pseudo-scientific information, 24.4% (n = 20) contained authoritative releases, and 75.6% (n = 62) contained normal personal releases. Most disinformation posts (n = 61 (74.3%)) were published from 16 February 2020 to 16 March 2020, and 12.2% (n = 10) were published from 1 February 2021 to 16 March 2021. Among the 44 dispelling disinformation posts, approximately 57.1% of the comments were in support, and 42.9% were opposed or invalid. Nearly half of the users were confused or superstitious about the disinformation.

**Conclusions:**

The disinformation about ACEI/ARB increasing the opportunity for COVID-19 infection during the pandemic was based on clinical mechanisms and scientific evidence intended for hypertensive patients taking long-term medication. It was packaged in a pseudo-scientific shell, leading to confusion and panic among patients. This disinformation harmed COVID-19 prevention efforts, damaged mental health, and possibly led to harmful behaviours. In future crises, the spread of rumours should be stopped quickly and effectively.

The outbreak of the coronavirus disease 2019 (COVID-19) pandemic [1] led to great social and economic burdens, with significant damage to both patients and health care systems. Mortality rates ranged from 2% to 10%, with increased risks of mortality and poor prognosis among elderly patients [2] and those with chronic diseases, including hypertension. Patients were affected not only by the risk of infection but also by ensuing stress and anxiety. Due to a lack of robust scientific evidence, there was a possibility of COVID-19-related disinformation spreading through online forums, blogs, and other online media.

COVID-19 uses angiotensin-converting enzyme type 2 (ACE-2) to introduce severe acute respiratory syndrome coronavirus 2 (SARS-CoV-2) into cells [3]. ACE-1 and its ACE-2 isozyme are two key angiotensin-converting enzymes of the renin-angiotensin system (RAS), which oppose ACE-1/angiotensin II (Ang II) pressor and tissue remodelling actions to regulate sodium homeostasis and blood pressure [4]. The renin-angiotensin-aldosterone system (RAAS) is usually activated under high blood pressure through the ACE-1-Ang II-AT1 receptor axis. ACE-2 combats RAS activation through the ACE-2/ANG(1-7)/MAS receptor pathway [5]. Angiotensin-converting enzyme inhibitors (ACEIs), which block the action of ACE-1, and angiotensin receptor blockers (ARBs), which block the action of angiotensin II at AT1 receptors, are two RAAS inhibitors widely used to treat hypertension, heart failure, and renal failure. Studies suggest that high expression of ACE-2 may increase susceptibility to infection [6], and ACEI or ARB treatment may increase ACE-2 expression [7]. This consequently led to misinformation spreading on how “ACEI/ARB will increase the concentration of ACE-2, thereby increasing the risk of infection and aggravating the disease after infection”. Hypertension is a primary risk factor for stroke, cardiovascular events, and chronic kidney disease, affecting approximately one-third of US adults [8], over 50% of individuals in Japan [9], and 25.2% of individuals in China. The global prevalence of hypertension is increasing as the population ages. ACEI/ARB are well-known not only for their effective anti-hypertensive properties and their role in preventing kidney damage caused by hypertension, but also for the improvement in ventricular remodelling and clinical outcomes in patients with diabetes and heart disease [10]. Consequently, they are now recommended as first-line therapy for patients with diabetes, albuminuria, and heart failure.

We investigated how disinformation about ACEI/ARB antihypertensive drugs and COVID-19 was disseminated on Sina Weibo, the largest Chinese microblogging platform, by employing text mining techniques to identify posts related to the taking of ACEI/ARB drugs and COVID-19 since the outbreak. According to official data, as of March 2020, Sina Weibo had more than 450 million users, highlighting its importance as a Chinese social media network. We aimed to analyse the content of these posts and public feedback to determine their truthfulness.

## METHODS

**Figure 1:**
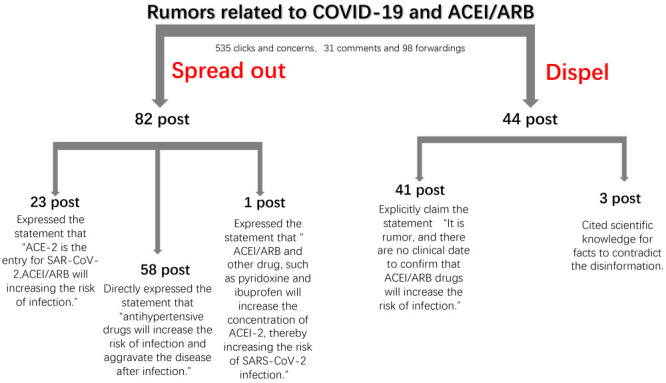
Post screening process and categories.

We used keywords such as “ACEI/ARB, COVID-19” or “antihypertensive drugs, COVID-19” to search for posts published on Sina Weibo between December 2019 and 19 January 2021 (**Figure 1**). We used text mining techniques to identify posts containing content related to the statement “antihypertensive drug ACEI/ARB can increase the risk of infection with COVID-19”. Two researchers screened the posts for content related to the statement “ACEI/ARB increase COVID-19 infection through increasing ACE-2 (with or without inhibit RAS)” or directly citing scientific literature, identifying them as pseudo-scientific disinformation. We descriptively analysed the compiled data set of disinformation.

## RESULTS

**Figure 2:**
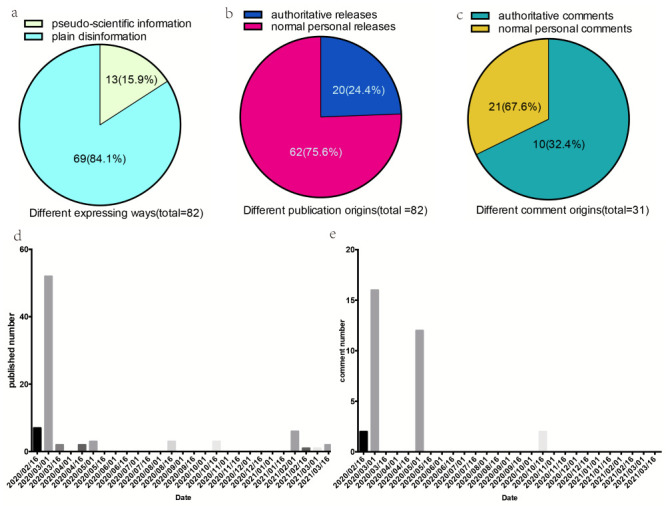
Structure of disinformation/comments and the change over time. **Panel A.** Structure depending on different ways in which comments were expressed. **Panel B.** Structure based on different publication origins. **Panel C.** Structure of comments based on different origins. **Panel D.** The change of publication number over time. **Panel E.** The change of comment number over time.

We identified 82 informative posts spreading disinformation and 44 dispelling this misinformation (**Figure 2**). Among the disinformation posts, 58 directly stated that “antihypertensive drugs will increase the risk of infection and aggravate the disease after infection”, 23 claimed that “ACE-2 is the entry for SARS-CoV-2, ACEI/ARB will increase the ACE-2 expression, thereby increasing the risk of infection”, and one stated that “ACEI/ARB and other drugs, such as pyridoxone and ibuprofen, will increase the concentration of ACE-2, thereby increasing the risk of SARS-CoV-2 infection”. The 82 disinformation posts received 535 clicks and concerns, 31 comments, and were forwarded 98 times. In contrast, the 44 posts that dispelled the disinformation included 41 posts that explicitly declared that “It is a rumor, and there are no clinical data to confirm that ACEI/ARB drugs will increase the risk of infection”. Three posts cited scientific knowledge or facts to contradict the disinformation.

We identified two types of disinformation in the 82 analysed posts based on their expression: pseudo-scientific information and plain disinformation. Pseudo-scientific information accounted for 13 (15.9%) of the posts and used technical terms such as ACE-2 expression, ACEI/ARB, and scientific research. The remaining 69 posts (84.1%) contained plain disinformation in more straightforward statements such as “antihypertension can increase the opportunity of COVID-19 infection”.

When considering their publication origin, 20 posts (24.4%) were authoritative releases, meaning they were published by persons with official titles or by companies or the industry. The other 62 (75.6%) were normal personal releases. Among the 31 analysed comments, 21 (67.6%) were authoritative comments and 10 (32.4%) were normal personal comments.

In analysing the changes in disinformation over time, we found most posts (n = 52 (63.4%)), were published on 1 March 2020. Furthermore, 61 (74.3%) of disinformation posts were published between 16 February and 16 March 2020, while 10 (12.2%) were published between 1 February and 16 March 2021. The remaining months had sporadic releases.

Dispelling disinformation did not always persuade users due to their inherent psychological and cognitive bias. We analysed public feedback to the 44 posts dispelling disinformation. Based on pre-determined classification criteria, two researchers screened the public comments and divided them into three categories: in support, opposed, and invalid. About 57.1% of comments were in support, while 42.9% were opposed or invalid. Unfortunately, nearly half of the users remained confused or superstitious regarding the disinformation.

## DISCUSSION

Researchers worldwide have raised concerns about the spread of rumours and disinformation on social media platforms. While these platforms were initially used to provide real-time updates and information on the pandemic, some media outlets, to gain social media clicks and attention, may have unintentionally misled the public during the early stages of the outbreak, when its transmission, pathophysiology, and treatment were not fully understood by scientists. We aimed to identify whether people believed that there was a link between ACEI/ARB and COVID-19 by analysing posts on Sina Weibo. We found 82 posts spreading disinformation and 44 posts dispelling it, while nearly half of the users were either confused or superstitious about the disinformation.

Public health authorities and governments could stop the spread of disinformation by taking down accounts spreading it, especially during the ongoing COVID-19 pandemic. Additionally, an authority figure with a large following may post against the rumour without using hashtags, as they increase the topic’s visibility with more users joining the discussion and using hashtags themselves.

ACE-2 is responsible for the cellular entry of SARS-CoV-2 by binding with the coronavirus spike protein [11]. Thus, the level of ACE-2 expression was initially believed to be a key risk factor for higher infection rates. It is now recognised that higher ACE-2 expression levels do not necessarily equate to a greater risk of infection. Claims that the use of ACEI/ARBs could increase the risk of infection and worsen the disease had caused panic and anxiety among hypertensive patients who use ACEI/ARB medication. Consequently, some users stopped taking their prescribed medication, leading to potentially severe cardiovascular adverse events and complications. However, reports show that patients with COVID-19 and hypertension have an increased risk of adverse outcomes, and those without antihypertensive treatment are associated with a significantly higher risk of mortality compared to those who are receiving antihypertensive treatments. Moreover, ACEI/ARBs appear to be associated with a lower risk of mortality [12]. The consequences of discontinuing RAAS inhibitors on cardiovascular risks and mortality among COVID-19 patients with hypertension remain unclear, so they must necessarily follow the instructions given by a medical professional regarding the continuation or discontinuation of ACEI/ARB medication. Bali et al. [13] found that rumours on how drinking salt water or bathing in it prevented Ebola resulted in 80% of patients exhibiting such behaviours before visiting a hospital during the Ebola epidemic, and that other similar rumours led to high-risk behaviour and a false sense of security.

The COVID-19 pandemic was the most serious global public health challenge in the past decade. Its high infectivity, mortality and morbidity rates, and the strict isolation measures established to prevent its transmission had generated widespread fear and anxiety in society. Despite health authorities and organisations publishing recommendations on COVID-19 information and patient education, significant improvements are still needed in patient education and stress management. The use of public media to share information increases during crises, making it difficult to identify disinformation. Furthermore, online virtual celebrities and influencers have a significant impact on the spread of information through their large number of followers, who may not necessarily verify the accuracy of this information. Consequently, a significant amount of disinformation, fake news, and related discussions have emerged and spread through online forums and blogs via new media channels. Overall, our findings emphasise the importance of combating disinformation related to health in online media platforms. It is essential that accurate, reliable information is disseminated to prevent confusion and fear among the public, especially during times of crises such as pandemics.

One limitation of our study is that we only included Chinese-language posts and only one platform. Additionally, our data collection method through several specific keywords may have excluded posts from users who did not use our target keyword or hashtag during the same period, leading to incomplete data collection. Future studies should use more advanced search strategies and classification methods, as well as search through other platforms and in other languages to obtain more complete data.

## CONCLUSIONS

During the COVID-19 pandemic, disinformation and fake news rapidly spread through social media, generating confusion, insecurity, and panic among the global population. This disinformation threatens individuals’ psychological well-being and can adversely affect public health. It can also lead to inappropriate health behaviours, especially among individuals with chronic illnesses who rely on long-term drug use. For instance, hypertensive patients who stop taking ACEI/ARB drugs are at greater risk for cardiovascular complications and mortality.

We analysed disinformation surrounding the use of ACEI/ARB drugs during the COVID-19 pandemic and its impact on hypertension patients. We identified key users and influential web sources and discussed potential strategies for combating this dangerous misinformation. We also found a lack of authority figures actively combating such misinformation. Policymakers should take efforts to isolate misinformation to avoid public health damage. Future research could further analyse data from Sina Weibo as the COVID-19 pandemic continues to evolve.
